# Sexual Dimorphism of the Gut Microbiota in the Chinese Alligator and Its Convergence in the Wild Environment

**DOI:** 10.3390/ijms232012140

**Published:** 2022-10-12

**Authors:** Meng-Yuan Hu, Qin-Zhang Yu, Jian-Qing Lin, Sheng-Guo Fang

**Affiliations:** 1 MOE Key Laboratory of Biosystems Homeostasis and Protection, State Conservation Center for Gene Resources of Endangered Wildlife, College of Life Sciences, Zhejiang University, Hangzhou 310058, China; 2 Guangdong Provincial Key Laboratory of Marine Disaster Prediction and Prevention, Guangdong Provincial Key Laboratory of Marine Biotechnology, Institute of Marine Science, Shantou University, Shantou 515063, China

**Keywords:** gut microbiota, serum metabolome, sexual dimorphism, Chinese alligator, *Alligator sinensis*, wildlife

## Abstract

The gut microbiota forms a complex microecosystem in vertebrates and is affected by various factors. As a key intrinsic factor, sex has a persistent impact on the formation and development of gut microbiota. Few studies have analyzed sexual dimorphism of gut microbiota, particularly in wild animals. We used 16S rRNA gene sequencing to analyze the gut microbiota of juvenile and adult Chinese alligators, and untargeted metabolomics to study serum metabolomes of adult alligators. We observed significant sexual differences in the community diversity in juvenile, but not adult, alligators. In terms of taxonomic composition, the phylum Fusobacteriota and genus *Cetobacterium* were highly abundant in adult alligators, similar to those present in carnivorous fishes, whereas the gut microbiota composition in juvenile alligators resembled that in terrestrial reptiles, indicating that adults are affected by their wild aquatic environment and lack sex dimorphism in gut microbiota. The correlation analysis revealed that the gut microbiota of adults was also affected by cyanobacteria in the external environment, and this effect was sex-biased and mediated by sex hormones. Overall, this study reveals sexual differences in the gut microbiota of crocodilians and their convergence in the external environment, while also providing insights into host–microbiota interactions in wildlife.

## 1. Introduction

Vertebrates have a wide variety of microbes, both internally and externally, that form a complex microecosystem, among which the most abundant and complex is the microbial community in the gastrointestinal tract [1,2]. Gut microbes and their hosts have evolved to form gut microbiota–animal mutualism [3,4]. The gut microbiota maintains homeostasis in the internal environment by extracting nutrients from food, regulating the immune system, and resisting pathogens [5,6,7], while the host provides the gut microbiota with a suitable environment, and nutrients for growth and reproduction [8]. The gut microbiota is consistently influenced by intrinsic factors, such as age and genetics, and is sensitive to multiple extrinsic factors, such as diet, temperature, and geographic location [9,10,11,12]. Among them, because of sex differences in behavior and physiology, sex as an important intrinsic factor leads to the differences of gut microbiota among individuals within species [13,14,15].

Sexual dimorphism in the gut microbiota of humans and mice has been well studied. Analysis of the Human Microbiome Project dataset showed that sex is correlated with the microbiota in the stool [16]. Independent analysis of 89 different inbred strains of mice showed significant differences in the gut microbiota between sexes of the same strain [17]. Correspondingly, multiple recent studies have demonstrated an association between gut microbiota and metabolic profiles. For example, specific gut microbes were correlated with specific metabolites in obese and diseased individuals [18,19]. Sex hormones, major metabolites responsible for sex dimorphism, are also associated with sex differences in the gut microbiota. The gut microbial composition of castrated adult male mice and Duroc pigs is more similar to that of females than that of uncastrated males [20,21]. Administering testosterone to castrated mice restored the gut microbiota to a certain extent [17], illustrating the impact of male hormones on gut microbiota. The gut microbiota, in turn, can modulate autoimmunity by altering sex hormone levels [13]. In addition, *Clostridium scindens*, a bacterium that converts glucocorticoids into androgens, has been found in the human gut [22]. However, most studies of sexual dimorphism in the gut microbiota have focused on humans and captive animals, and there is still a lack of equivalent studies in wild animals.

The Chinese alligator (*Alligator sinensis*), an endangered freshwater alligator endemic to China, is an ideal biological model for studying sexual dimorphism in the gut microbiota of wildlife. It has no sex chromosomes [23] and its sex is determined by its incubation temperature [24]. Research on sexual dimorphism in the gut microbiota of organisms with temperature-dependent sex determination and reptiles is lacking. Our study aims to fill this gap. Additionally, studies on the impact of sex on the gut microbiota of wildlife have focused on species with significant sexual dimorphism [25,26]. However, as a species with no obvious sexual dimorphism, it is worth exploring whether the gut microbiota of the Chinese alligator exhibits sex differences.

We used 16S rRNA gene sequencing to analyze the gut microbiota of juvenile (indoor captive-raised) and adult (wild-raised) Chinese alligators of both sexes. We also used untargeted metabolomic technology to identify metabolites in the serum of adult Chinese alligators and measured the correlation between gut microbiota and serum metabolites. This study is significant as it explores sexual dimorphism in the gut microbiota of Chinese alligators, and further analyses the interaction between the gut microbiota and serum metabolites.

## 2. Results

### 2.1. Composition and Relative Abundance of Microbiota

A total of 2,790,695 effective reads were obtained from 44 cloacal swab samples after quality control and chimera filtering. A total of 6381 OTUs were obtained, which were divided into 64 phyla, 148 classes, 330 orders, 483 families, and 856 genera. The rarefaction curves indicated that the sequencing depth was saturated (Figure S1 (Supplementary Materials)).

**Figure 1:**
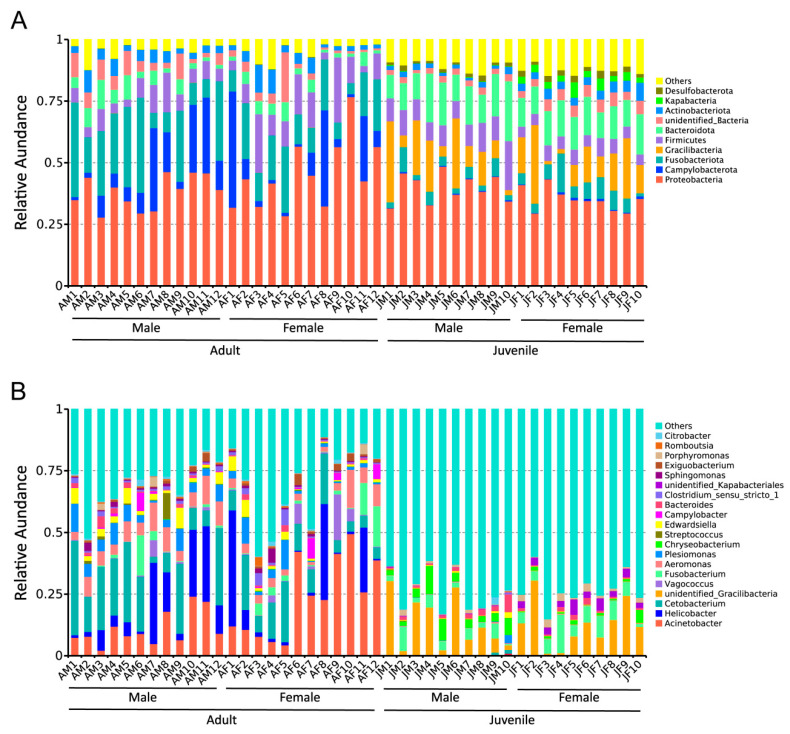
Relative abundance of the top 10 most abundant bacterial phyla (**A**) and the top 20 most abundant bacterial genera (**B**) in each sample. AM: adult male, AF: adult female, JM: juvenile male, JF: juvenile female.

Among the adult Chinese alligators, bacterial phyla with a mean relative abundance greater than 5% included Proteobacteria, Fusobacteriota, Campylobacterota, and Firmicutes, while in juvenile alligators such bacterial phyla included Proteobacteria, Bacteroidota, Gracilibacteria, Firmicutes, and Fusobacteriota (Figure 1A). Proteobacteria was the most abundant phylum in both alligator groups. Furthermore, Fusobacteriota, a core microbiota in both juvenile and adult alligators, is often enriched in the hindgut of carnivores [27,28], suggesting that the gut microbiota of the alligators is adapted to a carnivorous diet. The abundance of the other phyla, excluding Proteobacteria and Firmicutes, differed significantly between adult and juvenile Chinese alligators (Table S1).

At the genus level, *Acinetobacter* was the most dominant taxon in adult Chinese alligators, followed by *Cetobacterium*, *Helicobacter*, and *Aeromonas*. Each genus had a relative abundance greater than 5%. Unidentified *Gracilibacteria* was the dominant taxon in juvenile Chinese alligators, and the only other genus with relative abundance exceeding 5% was *Fusobacterium* (Figure 1B). The abundance of the top 20 genera differed significantly between adult and juvenile Chinese alligators (Table S2). *Cetobacterium* and *Fusobacterium* are representative microbial genera of the gut microbiota of aquatic carnivores [29,30,31] and terrestrial carnivores [27,28], respectively, suggesting that adult and juvenile Chinese alligators may be adapted to aquatic and terrestrial environments, respectively.

### 2.2. Sex and Age-Biased Community Diversity

**Figure 2:**
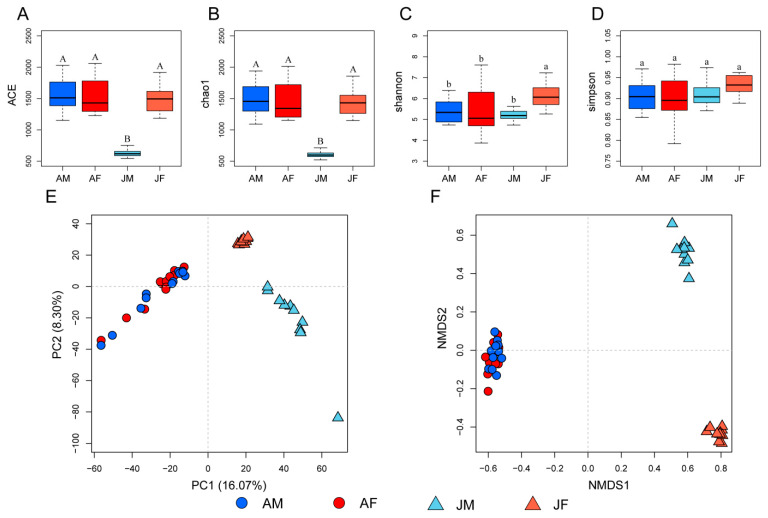
Community diversity of gut microbiota in Chinese alligators. Alpha diversity of gut microbial communities in each group illustrated using ACE (**A**), chao1 (**B**), Shannon (**C**), and Simpson (**D**) indices. Comparing beta diversity using principal component analysis (PCA) (**E**) and non-metric multi-dimensional scaling (NMDS) (**F**). 0.01 < *p* < 0.05 are marked with lowercase letters and *p* < 0.001 are marked with uppercase letters. AM: adult male; AF: adult female; JM: juvenile male; JF: juvenile female.

We analyzed the alpha diversity of gut microbiota in both male and female juvenile and adult Chinese alligators by computing four diversity indices (Figure 2A–D). ACE and chao1 indices showed that the community richness of the gut microbiota in male juveniles was significantly lower than in the other three groups (Wilcoxon rank-sum test, *p* < 0.001), and there were no significant differences between the other groups (Figure 2A,B). The Shannon index, a measure of community richness and evenness, showed that the diversity of the gut microbial community of juvenile females was significantly higher than that of the other three groups (Wilcoxon rank-sum test, *p* < 0.05), and there were no significant differences between the other groups (Figure 2C). Although the juvenile female group had a higher Simpson index than the other three groups, this difference did not reach statistical significance (Figure 2D). These results indicate significant differences in the richness and diversity of the gut microbial community between sexes in juvenile but not in adult Chinese alligators.

To analyze beta diversity, we used PCA and NMDS to assess differences in the community structure between groups (Figure 2). Adult males and females were clustered together, whereas juvenile males and females were clustered separately from each other and from the adults. This illustrates that the structure of the gut microbiota community differs significantly between sexes in juvenile Chinese alligators, but this community structure changes significantly and sexually converges in the adults. PC1 and NMDS1 axes were dominated by differences between juvenile and adult alligators, while PC2 and NMDS2 revealed sexual differences between juvenile alligators (Figure 2E,F). The ADONIS analysis gave comparable results. There were significant differences between juveniles and adults (R^2^ = 0.67, *p* < 0.001 for males and R^2^ = 0.61, *p* < 0.001 for females) and between sexes in the juvenile groups (R^2^ = 0.26, *p* < 0.001). However, the differences between adult males and females were less obvious (R^2^ = 0.11, *p* = 0.028). These results point to the sexual dimorphism of the gut microbiota in juvenile Chinese alligators and their convergence in adults.

### 2.3. Sex and Age-Biased Taxonomic Composition

LEfSe was used to analyze microbial taxa that differed between sexes (Figure S2). A total of 15 taxa differed between adult male and female groups, including 12 male-biased and 3 female-biased taxa. The main taxa that contributed to sex differences in adult Chinese alligators were the orders Enterobacterales and Pseudomonadales, the family Moraxellaceae, and the genus *Acinetobacter* (Figure S2A). Additionally, 19 taxa were significantly different between juvenile males and females, including 12 male-biased and 7 female-biased taxa. The phyla Firmicutes and Kapabacteria and the orders Flavobacteriales and Kapabacteriales were the main taxa responsible for sex differences between juvenile Chinese alligators (Figure S2B). Significant differences between juvenile males and females were also observed in the kingdom bacteria, with gut bacteria being more abundant in males than in females. We also used the Wilcoxon rank-sum test to compare the microbial taxa (phylum and genus) with significant differences between the sexes (Tables S3–S5). No microbial taxa differed significantly at the phylum level between adult males and females, whereas 41 taxa differed significantly at the genus level (Table S4). In comparison, there were 34 and 304 taxa with significant differences at the phylum and genus levels, respectively, between juvenile males and females (Tables S3 and S5). These results are consistent with those of community diversity, that is, sex differences in the gut microbiota of juvenile Chinese alligators were more significant than those of gut microbiota in adult Chinese alligators.

Age-biased LEfSe analysis identified 55 taxa that were significantly different between male adults and juveniles, including 30 adult-biased and 25 juvenile-biased taxa (Figure S3A), and 52 taxa that were significantly different between female adults and juveniles, including 30 adult-biased and 22 juvenile-biased taxa (Figure S3B). Additionally, the Wilcoxon rank-sum test identified significant differences in the abundances of 35 phyla and 385 genera between male adults and juveniles (Tables S6 and S7), and 26 phyla and 422 genera between female adults and juveniles (Tables S8 and S9). These results indicate that age differences were greater than sex differences in the gut microbiota of Chinese alligators.

### 2.4. Analysis of Differential Metabolites between Adult Male and Female Chinese Alligators

LC-MS was used to perform untargeted metabolomics on serum metabolites of adult alligators to explore gut microbiota–host interactions in Chinese alligators. A total of 894 metabolites were detected and quantified, including 558 in the positive ion mode and 336 in the negative ion mode. The identified metabolites were annotated using information in KEGG (Figure S4), HMDB (Figure S5), and LIPID MAPS databases (Figure S6).

**Figure 3:**
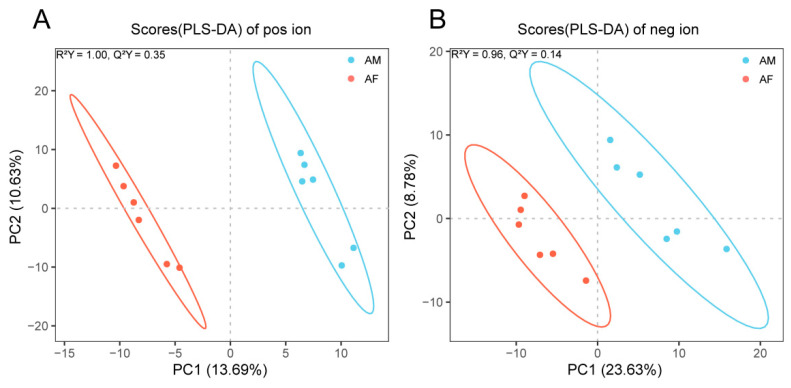
Partial least squares discriminant analysis (PLS-DA) score plots displaying the discrimination of serum metabolomes between adult male and female Chinese alligators in positive (**A**) and negative (**B**) ion modes based on the first two principal components (PCs). AM: adult male; AF: adult female.

**Figure 4:**
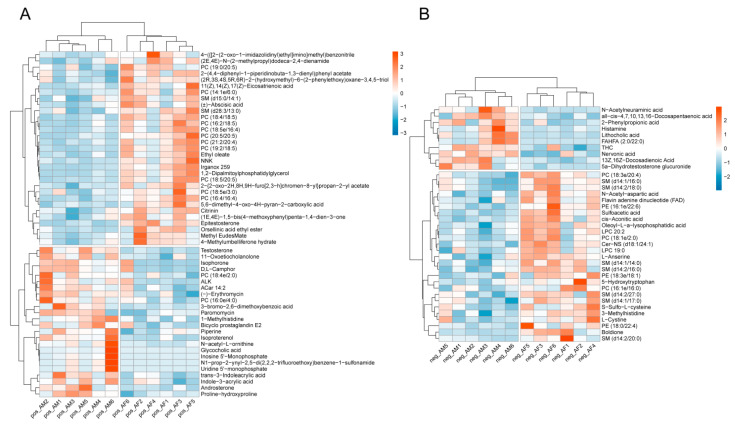
Hierarchical clustering heatmaps of differential metabolites between adult male and female Chinese alligators in positive (**A**) and negative (**B**) ion modes. AM: adult male; AF: adult female.

PLS-DA was used to characterize differences in global serum metabolomics between male and female adult alligators (Figure 3). PLS-DA score plots showed that serum metabolomes of adult male and female Chinese alligators differed significantly under both positive (Figure 3A) and negative (Figure 3B) ion modes. Subsequent screening of differential metabolites identified 55 and 37 metabolites with significant differences (VIP > 1.0, FC > 1.2 or FC < 0.833, and *p* < 0.05) under positive and negative ion modes, respectively. Hierarchical clustering heatmaps of differential metabolites also showed that samples from the six male and six female alligators clustered together by sex (Figure 4). In the positive ion mode, 24 metabolites were enriched in adult males, including two androgens—testosterone and androgen—and 31 metabolites were enriched in adult females, including epitestosterone (Figure 4A). In the negative ion mode, 10 metabolites were enriched in adult males, including 5α-dihydrotestosterone glucuronide, and 27 metabolites were enriched in adult females, including boldione (Figure 4B).

We conducted a KEGG pathway enrichment analysis to explore the biological functions of these differential metabolites (Figure S7). In the positive ion mode, KEGG analysis revealed that two differential and ten non-differential metabolites were enriched in the steroid hormone biosynthesis pathway (Figure S7A). We simplified this pathway in KEGG and constructed the androgenic hormone biosynthesis pathway (Figure S8).

### 2.5. Analysis of Correlations between Gut Microbiota and Serum Metabolome

**Figure 5:**
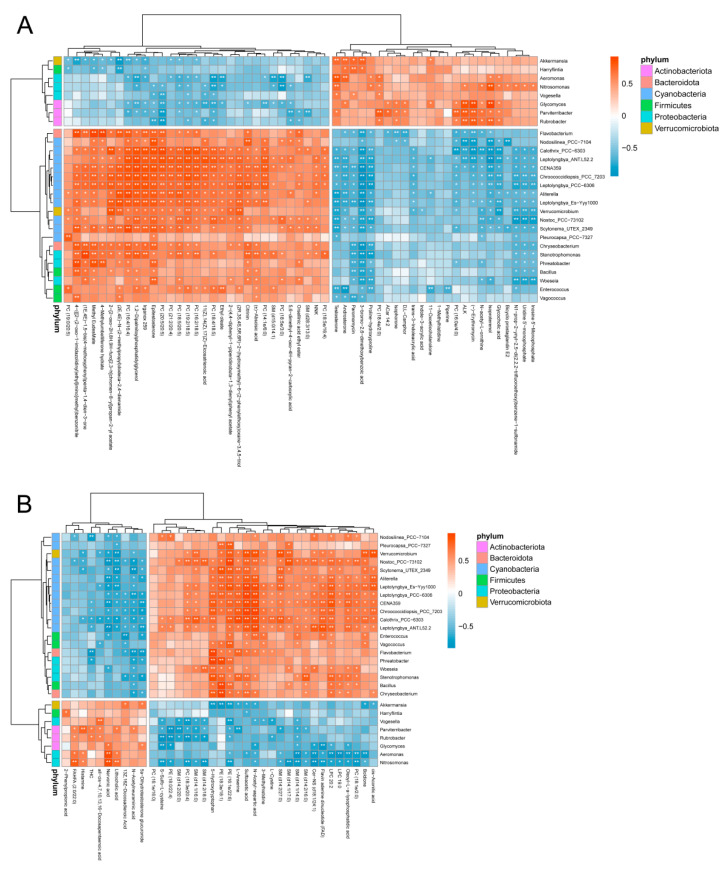
Integrated correlation analysis of sex-biased microbes and metabolites in adult Chinese alligators. Heatmap of Spearman’s rank correlation coefficients of metabolites from positive (**A**) and negative (**B**) ion modes. The shade of color indicates the magnitude of the correlation coefficient (red and blue indicate positive and negative correlations, respectively). Significant correlations are indicated by white stars: * *p* < 0.05, ** *p* < 0.01.

We used Spearman’s correlation analysis to evaluate the relationship between sex-biased bacterial genera and sex-biased metabolites (Figure 5). The sex-biased genera that were most highly correlated with metabolites mostly belonged to the phylum cyanobacteria and included *Nostoc_PCC−73102*, *Scytonema_UTEX_2349*, *Aliterella*, *Leptolyngbya_Es−Yyy1000*, *Chroococcidiopsis_PCC_7203*, *Calothrix_PCC−6303*, *CENA359*, *Leptolyngbya_PCC−6306*, and *Leptolyngbya_ANT.L52.2*. These genera were all enriched in the gut of adult female alligators and were negatively correlated with androsterone and testosterone, and positively correlated with epitestosterone. This implies that males have better resistance to the disturbance caused by cyanobacteria in the external environment, and this resistance may be mediated by androgen.

## 3. Discussion

Studying the intrinsic factors affecting the gut microbiota of wild animals is often difficult because their gut microbiota is affected by multiple extrinsic factors [11,25]. Here, we explored the sex dimorphism in the gut microbiota of the Chinese alligator and its possible causes by comparing gut microbiota and serum metabolites based on sex and age and analyzing the correlation between sex-biased bacterial genera and sex-biased metabolites. To our knowledge, this is the first study to analyze sex differences in the gut microbiota of reptiles and the first to analyze the interaction between gut microbiota and serum metabolites in these animals.

The core gut microbial communities of adult Chinese alligators were dominated by Proteobacteria, Fusobacteriota, Campylobacterota, and Firmicutes. A previous study on Chinese alligators mainly focused on differences in the gut microbiota between hibernation and active periods, and the microbiota in the colon and feces during the active period was similar to that of adult Chinese alligators in this study; however, the biggest difference is that phylum Campylobacterota was not detected in the previous study [32]. This difference may be due to the fact that bacteria in the phylum Campylobacterota previously belonged to the phylum Proteobacteria, known as class Epsilonproteobacteria [33,34]. Comparing adult Chinese alligators with other species showed that their gut microbiota had adapted to their habitat. In general, the gut microbial communities of most reptiles, including lizards [35,36], snakes [37,38], and turtles [39,40], are dominated by varying proportions of Proteobacteria, Firmicutes, and Bacteroidetes. However, in this study, the phylum Fusobacteriota, previously called Fusobacteria [41], was one of the core gut microbial communities in adult Chinese alligators, consistent with previous studies of the Chinese alligator, the American alligator (*Alligator mississippiensis*), and the Siamese crocodile (*Crocodylus siamensis*) [32,42,43]. Fusobacteriota is commonly enriched in the hindgut of carnivores, including the northern elephant seal (*Mirounga angustirostris*) [25], tiger (*Panthera tigris*) [27], black vulture (*Coragyps atratus*) [28], tropical gar (*Atractosteus tropicus*) [44], and Southern catfish (*Silurus meridionalis*) [45], suggesting that the gut microbiota of the adult Chinese alligator has distinct carnivorous features. In addition, *Cetobacterium*, the most common genus in the phylum Fusobacteriota, was the most abundant genus in the guts of adult male Chinese alligators. This genus is also enriched in the guts of various fishes [29,30,31]. The habitats and diets of these fishes are similar to those of adult Chinese alligators, implying that the adaptation of adult Chinese alligators to the aquatic environment and piscivorous diet makes their gut microbiota more similar to those of carnivorous fishes than to those of terrestrial reptiles.

In contrast, the core gut microbiota of juvenile Chinese alligators was dominated by Proteobacteria, Bacteroidetes, Gracilibacteria, Firmicutes, and Fusobacteriota. The abundance of Fusobacteriota was significantly lower in the guts of juvenile Chinese alligators than in the guts of adult Chinese alligators (5.35% vs. 18.61%, *p* < 0.001), and the most abundant genus in this phylum was *Fusobacterium* rather than *Cetobacterium*, which is consistent with many terrestrial carnivores [27,28]. This phenomenon may indicate that juvenile Chinese alligators in captivity have not yet begun to adapt to the aquatic environment in the wild, and their gut microbiota maintains a terrestrial state.

We observed no significant sex differences in the richness and diversity of the gut microbiota community in adult Chinese alligators but found significant sex differences in both community richness and the Shannon index of community diversity in juvenile Chinese alligators. Additionally, female juvenile Chinese alligators had significantly higher community diversity compared with male juveniles, consistent with previous studies in human infants and adults, mice, and Duroc pigs [20,21,46,47]. The northern elephant seal is different, however, with males exhibiting significantly higher alpha diversity than females, although the study in question suggested that a higher proportion of healthy male individuals are responsible for this result [25]. As for beta diversity, the gut microbiota of juvenile Chinese alligators showed significant sex differences based on both PCA and NMDS results. Although the gut microbiota of adult Chinese alligators did not show significant sex differences in community diversity, the ADONIS test identified significant sex differences in community structure. This is the first report of sex dimorphism in reptilian gut microbiota and shows that these sex differences are found primarily in juvenile alligators. Previous studies showed that the vast majority of sex dimorphisms in gut microbiota occur in adults, with sex dimorphism in the gut microbiota of juveniles being found only in humans and northern elephant seals [25,46].

We used both LEfSe and Wilcoxon rank-sum test to identify sex-biased microbial taxa. No sex-biased microbial taxa were identified at the phylum level in adult Chinese alligators, consistent with the results from the analysis of community diversity. The three sex-biased microbial genera with the greatest abundance in the adult Chinese alligators were all pathogenic bacteria: *Acinetobacter*, *Aeromonas*, and *Plesiomonas* [48,49,50], and belonged to the top ten genera identified in the adults. However, the abundance of these genera in the guts of juvenile Chinese alligators was low, suggesting that these bacteria may not be native to the alligator but come from the wild environment. A pathogenicity study showed that female mice were more susceptible following infection with *Acinetobacter baumannii*, while the susceptibility of male mice treated with estradiol also increased [51]. Likewise, although adult Chinese alligators of both sexes are affected by external pathogenic bacteria, the differences in their immune responses result in sex-biased differences in the abundance of these pathogenic bacteria. In juvenile Chinese alligators, 34 sex-biased bacterial phyla and 304 sex-biased bacterial genera were identified using the Wilcoxon rank-sum test, while only 19 sex-biased bacterial taxa were identified using LEfSe, likely due to the greater uniformity in the gut microbial community of juvenile Chinese alligators, resulting in the LDA scores of some taxa not reaching the required threshold. Many sex-biased microbial phyla and genera identified in the guts of juvenile Chinese alligators were absent in the guts of male juvenile Chinese alligators, consistent with the lower diversity of gut microbial communities in males. The most abundant sex-biased microbial phylum in juvenile alligators was Firmicutes, whose most notable bacterial genus is the male-biased *Lactobacillus*. *Lactobacillus* can promote host health by inhibiting the colonization of pathogenic bacteria by occupying functional niches [52], producing anti-pathogenic compounds [53], enhancing epithelial cell-barrier function [54], and modulating host immune-responses [55]. Therefore, the low diversity observed in the gut microbial community of juvenile, male Chinese alligators may be associated with higher abundance of Lactobacillus.

We extracted blood from adult Chinese alligators, but not from juvenile Chinese alligators due to the potential for excessive damage, and performed serum metabolomic analysis to explore gut microbiota–host interactions. A total of 92 sex-biased metabolites were identified between adult males and females, which included steroid hormones such as male-biased testosterone and androsterone and female-biased epitestosterone. Differences in immune responses between sexes are largely regulated by sex hormones [56], with androgens playing an immunosuppressive role [57]. The gastrointestinal tract, the largest immune organ in the body, is also regulated by androgen-related immunoendocrine [58]. The gut microbiota of castrated male mice and pigs are more closely related to those of females than to those of uncastrated males [20,21], while the gut microbiota of castrated male mice is restored to some extent after administration of testosterone [17], suggesting that androgens have dramatic effects on gut microbial communities. The serum metabolomes of adult Chinese alligators showed significant sex differences, and the gut microbiota of these alligators is expected to have corresponding sex differences. However, we did not observe these differences in this study. As we mentioned above, the gut microbiota of adult Chinese alligators differs from that of terrestrial reptiles but is more similar to that of aquatic carnivorous fish, and a variety of aquatic pathogenic bacteria have been found in their guts. Therefore, we believe that the contents of the gut microbiota of adult Chinese alligators are influenced by their aquatic environment, and these environmental factors mask the sex differences.

Correlation analysis showed the relationship between serum metabolites and gut bacteria in adult Chinese alligators. Female-biased genera belonging to Cyanobacteria were highly correlated with multiple metabolites, including testosterone, androsterone, and epitestosterone. Previous studies have shown that exposure to cyanobacteria alters the liver metabolome of multiple freshwater fish [59,60]. Furthermore, exposure of medaka fish (*Oryzias latipes*) to cyanobacteria has a more pronounced effect on the liver metabolome and proteome in females than in males [61,62]. In this study, the gut microbiota of adult Chinese alligators had a significantly higher proportion of cyanobacteria compared with that of juvenile alligators, suggesting that the living environments of adult alligators may be rich in the cyanobacteria that affect both the gut microbiota and the serum metabolome of the Chinese alligators.

In this study, we analyzed the gut microbiota of juvenile and adult Chinese alligators, but to avoid harm to juveniles, we only analyzed the serum metabolome of adults. In subsequent studies, the data on serum metabolome of juvenile Chinese alligator need to be supplemented. At the same time, additional sampling at more ages from birth to adulthood could be added to observe how the sex differences in gut microbiota and serum metabolome change with age. In addition, it is also a direction for future research to select several released Chinese alligators and put them back in captivity to observe whether the sex differences in gut microbiota will reappear.

## 4. Materials and Methods

### 4.1. Microbiota and Metabolite Sampling

Cloacal swabs and serum samples were collected from Chinese alligators at the Changxing Yinjiabian Chinese Alligator Nature Reserve in May 2021. The Changxing Reserve is an extension of the original habitat of the Chinese alligator, especially the wild release area, which has largely restored the habitat of the Chinese alligator. So far, more than 2000 Chinese alligators have adapted to the wild environment in the wild release area. Prior to sampling, the 7-year-old adult male (AM, *n* = 12) and female (AF, *n =* 12) Chinese alligators lived in the wild environment of the reserve and preyed on their own (mainly various fish species), while the 2-year-old juvenile male (JM, *n =* 10) and female (JF, *n =* 10) Chinese alligators were raised in a greenhouse at the reserve and were fed the same food (minced fish). The gut microbiota of these 44 Chinese alligators was sampled using cloacal swabs and stored in sterile preservation tubes. This non-invasive method of sampling the gut microbiota of reptiles is generally accepted [43,63]. Since blood extraction from Chinese alligators is not easy and will cause certain damage, we only selected 12 individuals (six females and six males) from the 24 above-mentioned adult Chinese alligators for blood extraction. Blood samples were extracted from the caudal vein of the tail and centrifuged at 2000 rpm and 4 °C for 10 min to obtain serum samples, which were then stored at –80 °C until further use.

### 4.2. Amplicon Sequencing

Total DNA was extracted from the cloacal swab samples using the cetyltrimethyl ammonium bromide (CTAB) method. The V3–V4 region of the bacterial 16S rRNA gene was amplified using the primers 341f (5′-CCTAYGGGRBGCASCAG-3′) and 806r (5′-GGACTACHVGGGTWTCTAAT-3′). Each PCR reaction consisted of 15 µL of Phusion^®^ High-Fidelity PCR Master Mix (New England Biolabs, Ipswich, Massachusetts, USA), 2 µM of forward and reverse primers, and about 10 ng template DNA. The TruSeq DNA PCR-Free Sample Preparation Kit (Illumina, San Diego, CA, USA) was used for library construction. The prepared library was sequenced using NovaSeq 6000 (Illumina) and 250 bp paired-end reads were generated.

### 4.3. Sequence Data Analysis

Sequencing reads were merged using FLASH (V1.2.11, http://ccb.jhu.edu/software/FLASH/, accessed on 21 June 2021) [64]. Raw tags were filtered with QIIME (V1.9.1, http://qiime.org/scripts/split_libraries_fastq.html, accessed on 21 June 2021) [65] under specific filtering conditions [66] to obtain clean tags. The UCHIME algorithm (UCHIME, http://www.drive5.com/usearch/manual/uchime_algo.html, accessed on 22 June 2021) [67] was used to locate these tags in the Silva database [68], and chimera sequences were removed to obtain effective tags. Uparse algorithm (Uparse V7.0.1001, http://www.drive5.com/uparse/, accessed on 22 June 2021) [69] was used to cluster effective tags with 97% identity into the same operational taxonomic units (OTUs). Taxonomic information for each representative OTU was annotated using the SSUrRNA database of SILVA138 (http://www.arb-silva.de/, accessed on 25 June 2021) based on the Mothur algorithm (Mothur V1.43.0, https://mothur.org/, accessed on 25 June 2021) [70]. The sample with the fewest sequences was used as the standard to normalize OTU abundance.

Alpha diversity was used to analyze the complexity of species diversity by six indicators (Observe-species, Chao1, Shannon, Simpson, ACE, and Good coverage). All these indices in our samples were calculated using QIIME and visualized using R software v4.0.5, Vienna, Austria. To determine beta diversity, QIIME software was used to calculate the Unifrac distance, and R software v4.0.5 was used to perform principal component analysis (PCA) and non-metric multi-dimensional scaling (NMDS). The R package vegan was used for ADONIS analysis, based on the Bray–Curtis distance matrix. The Wilcoxon rank-sum test was used for pairwise comparisons of diversity index and taxonomic composition. The LEfSe software [71] was used to identify biomarkers that differed significantly between study groups (*p* < 0.05 and linear discriminant analysis (LDA) score > 4.0).

### 4.4. Metabolite Extraction and Detection

Untargeted metabolomic analysis was performed on 12 serum samples from adult Chinese alligators (six males and six females). Each 100 μL serum sample was resuspended in pre-cooled 80% methanol and 0.1% formic acid via vortexing. The suspension was incubated on ice for 5 min and then centrifuged at 15,000× *g* and 4 °C for 20 min. Some of the supernatant was diluted with liquid chromatography–mass spectrometry (LC-MS) grade water to obtain a solution containing 53% methanol. The supernatant was centrifuged at 15,000× *g* and 4 °C for 20 min and then injected into the LC-MS/MS system for analysis [72].

UHPLC-MS/MS analyses were performed at Novogene Co., Ltd. (Beijing, China) using a Vanquish UHPLC system (Thermo Fisher Scientific, Waltham, MA, USA) coupled with an Orbitrap Q Exactive HF-X mass spectrometer (Thermo Fisher Scientific, Waltham, MA, USA). Briefly, samples were injected into a Hypesil Gold column (100 × 2.1 mm, 1.9 μm) at a flow rate of 0.2 mL/min using a 17-min linear gradient. Eluents A (0.1% FA in water) and B (Methanol) were used in the positive mode, while eluents A (5 mM ammonium acetate, pH 9.0) and B (Methanol) were used in the negative mode. The Q Exactive HF-X mass spectrometer was operated in positive/negative polarity modes with spray voltage of 3.2 kV, a capillary temperature of 320 °C, sheath gas flow rate of 40 arb, and aux gas flow rate of 10 arb.

### 4.5. Metabolite Identification and Analysis

Raw data files were processed using the Compound Discoverer v3.1 (Thermo Fisher Scientific, Waltham, MA, USA) to perform peak alignment, peak picking, and quantitation for each metabolite. Subsequently, peak intensities were normalized to the total spectral intensity. Normalized data were used to predict the molecular formula based on additional ions, molecular ion peaks, and fragment ions, and then matched against data in mzCloud (https://www.mzcloud.org/, accessed on 2 July 2021), mzVault, and MassList databases to obtain accurate qualitative and relative quantitative results.

The Kyoto Encyclopedia of Genes and Genomes (KEGG), human metabolome database (HMDB), and LIPID maps databases were used to annotate the metabolites. Partial least squares discriminant analysis (PLS-DA) was performed in mataX. Statistical significance was calculated using the *t*-test. Metabolites with variable importance in the projection (VIP) > 1, *p* < 0.05, and Fold Change (FC) ≥ 1.2 or FC ≤ 0.833 were considered differential metabolites. Enriched metabolic pathways were identified using KEGG pathway enrichment analysis. Metabolic pathways with *p* < 0.05 were considered significantly enriched.

### 4.6. Spearman Correlation Analysis

Bacterial genera that differed significantly between groups of microbial samples corresponding to metabolome samples were identified using the Wilcoxon rank-sum test. Spearman correlation analysis of sex-biased genera and sex-biased metabolites was performed using the R package psych. Significant correlations were represented by *p* < 0.05. Finally, a correlation heatmap was constructed using the R package pheatmap.

## 5. Conclusions

We found significant sex dimorphism in the gut microbiota of juvenile, but not adult, Chinese alligators. These differences were attributed to the influence of the external environments. To our knowledge, this is the first study to identify sex-biased differences in the gut microbiota of reptiles and shows that sex differences in gut microbiota also occur in species that do not have apparent morphological sex dimorphism. Our analysis of host–microbiota interactions also showed that sex hormones are associated with bacterial genera in the phylum Cyanobacteria. Overall, our study reveals sexual dimorphism in the gut microbiota of alligators, emphasizes the masking effect of the external environment on internal factors, and provides new insights into host–microbiota interactions in wild animals.

## Data Availability

Raw sequencing data generated in this study have been deposited in the NCBI database under the accession number PRJNA835985. Raw metabolomics datafiles have been deposited in the EMBL-EBI MetaboLights database with the identifier MTBLS4841.

## Supplementary Materials

The following supporting information can be downloaded at: https://www.mdpi.com/article/10.3390/ijms232012140/s1.
