# Supplementary material for: Sexual Dimorphism of the Gut Microbiota in the Chinese Alligator and Its Convergence in the Wild Environment
Source: Int J Mol Sci. 2022 Oct 12;23(20):12140. doi: 10.3390/ijms232012140 (PMC9603114; doi:10.3390/ijms232012140)
Supplement: Supplementary file 1 [file ijms-23-12140-s001.zip › ijms-1948675-supplementary.pdf]

Supplementary Figures

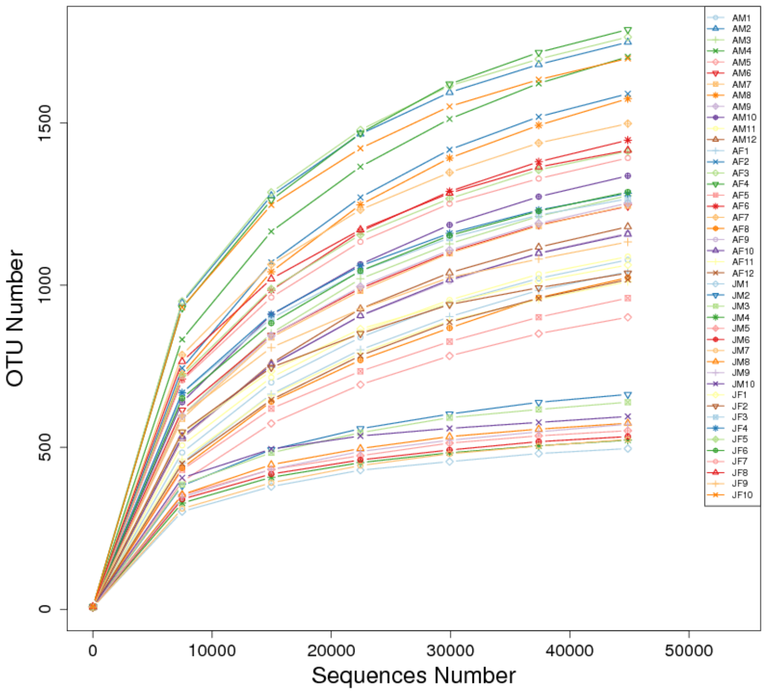

Figure S1. The rarefaction curve for each sample.

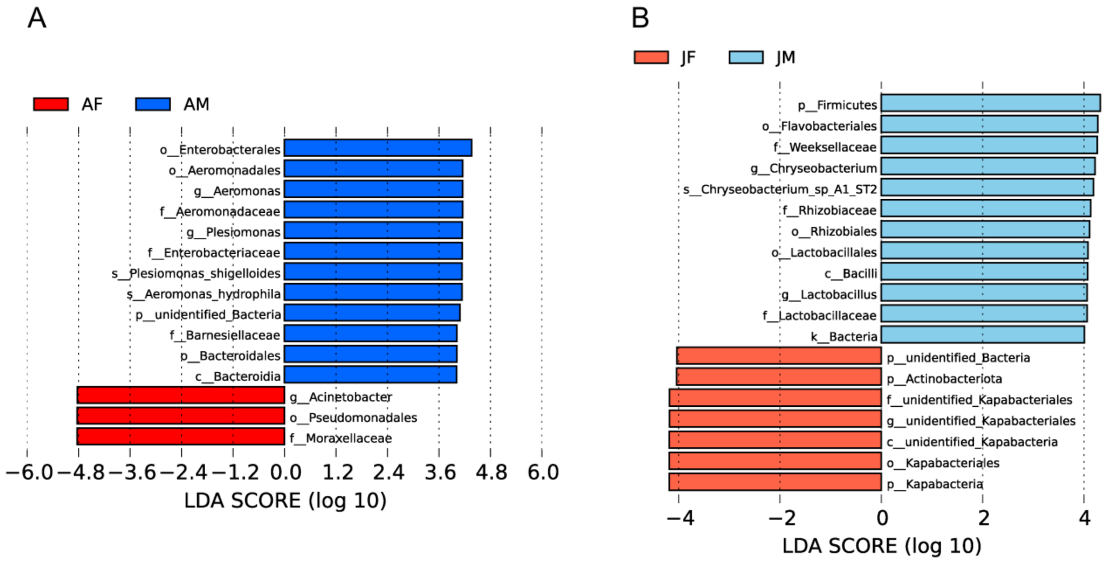

Figure S2. Bacterial taxa with significant sex-biased differences in adult (A) and juvenile (B) Chinese alligators identified using linear discriminant analysis effect size (LDA > 4, $p < 0.05$). AM: adult male, AF: adult female, JM: juvenile male, JF: juvenile female.

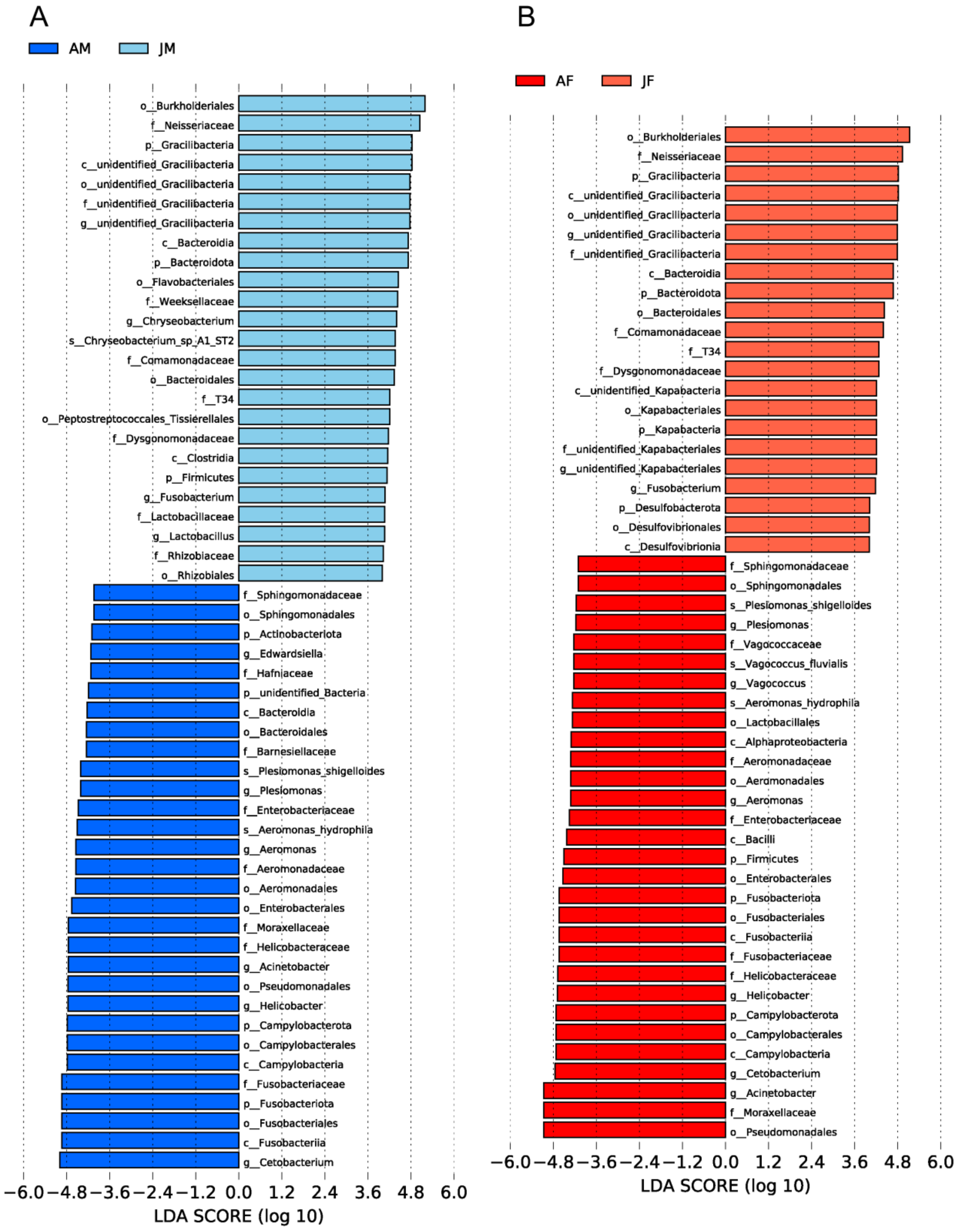

Figure S3. Bacterial taxa with significant age-biased differences in male (A) and female (B) Chinese alligators identified using linear discriminant analysis effect size (LDA > 4, $p < 0.05$). AM: adult male, AF: adult female, JM: juvenile male, JF: juvenile female.

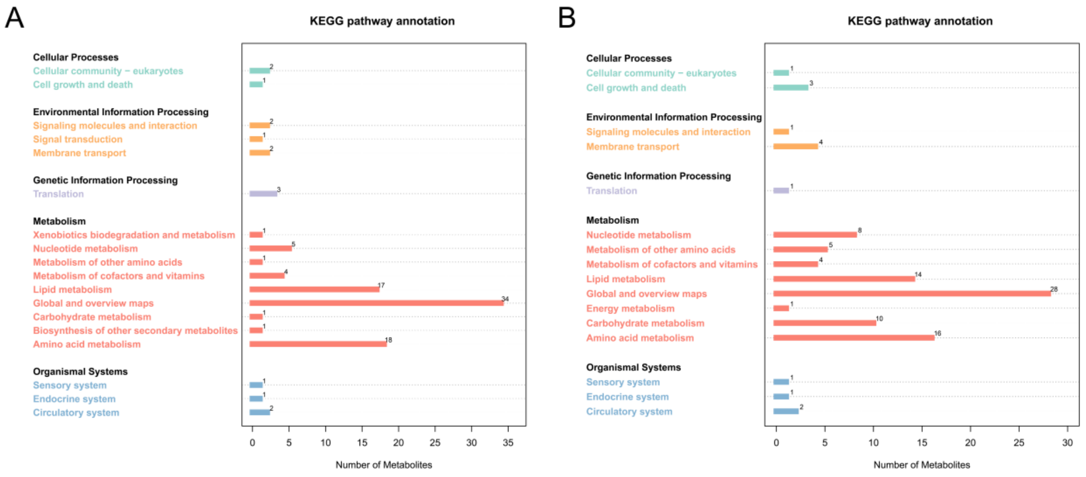

Figure S4. Annotation results of metabolites in positive ion mode (A) and negative ion mode (B) in KEGG database.

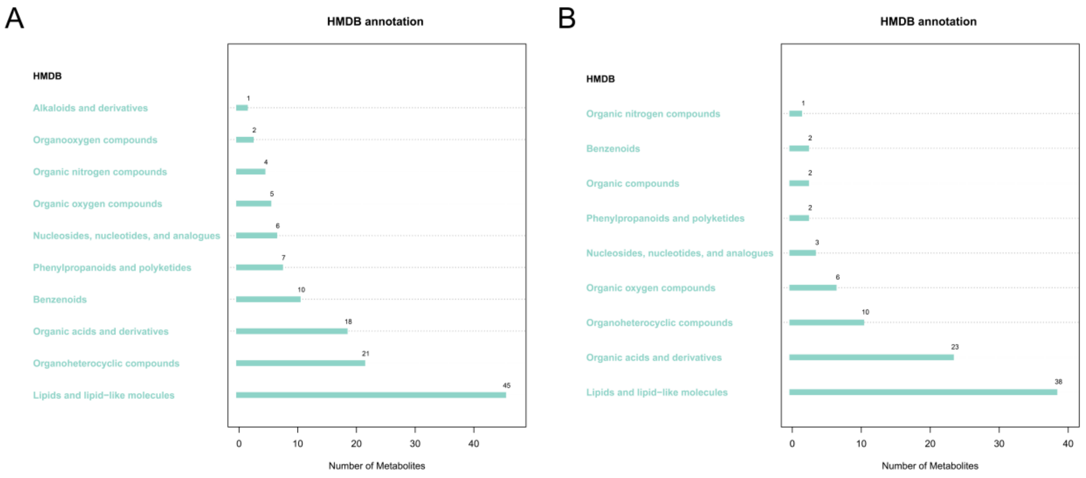

Figure S5. Annotation results of metabolites in positive ion mode (A) and negative ion mode (B) in HMDB database.

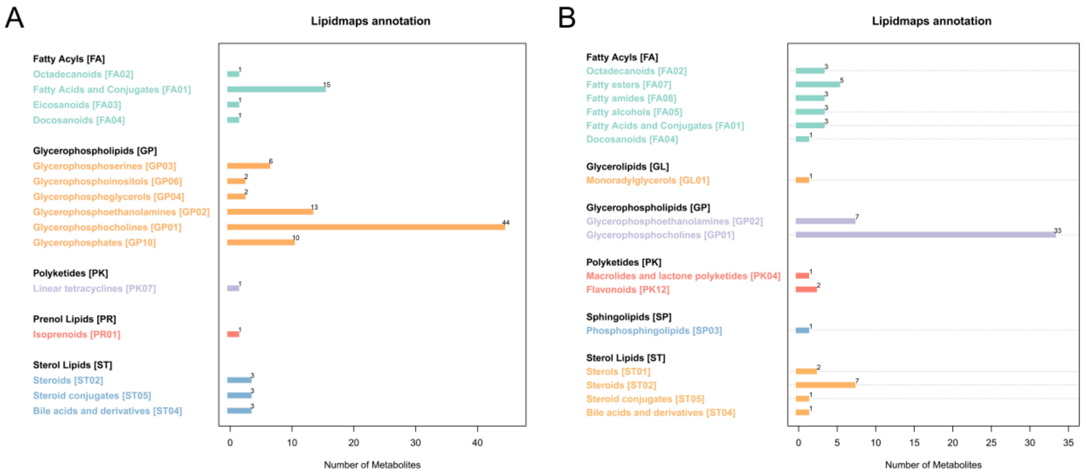

Figure S6. Annotation results of metabolites in positive ion mode (A) and negative ion mode (B) in LIPID MAPS database.

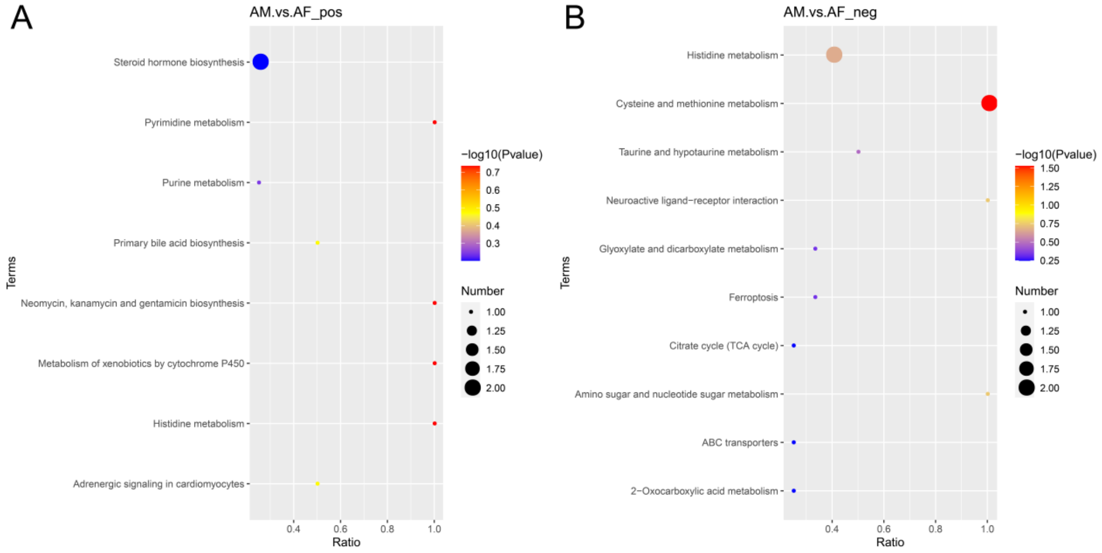

Figure S7. Enriched KEGG pathways for sex-biased serum metabolites in positive (A) and negative (B) ion modes.

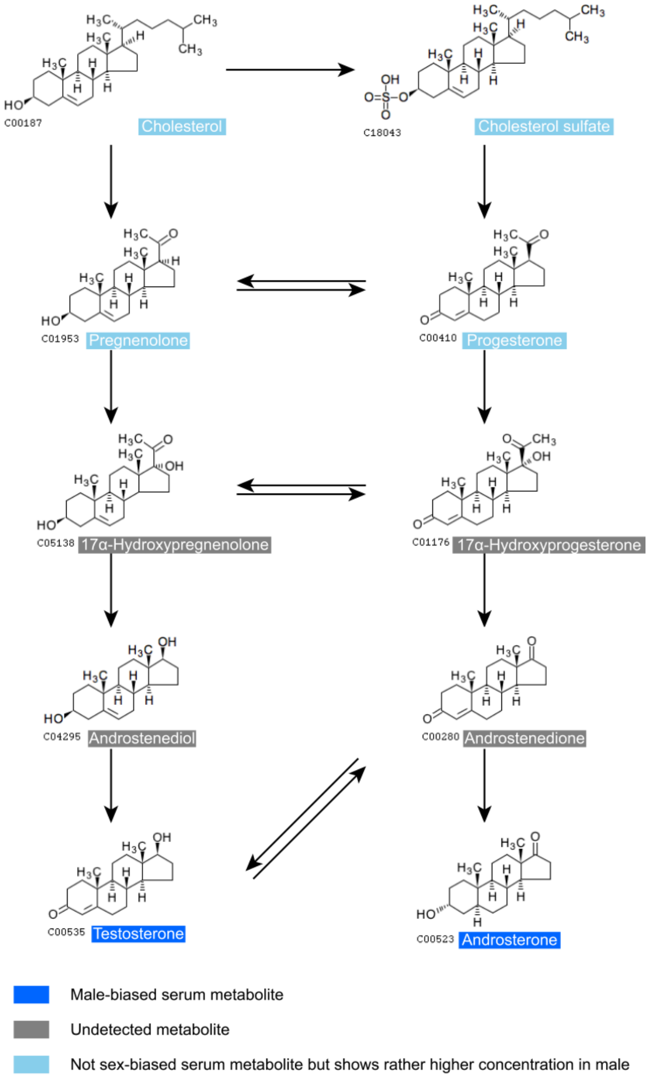

Figure S8. Sex-biased patterns of metabolites in the androgenic hormone biosynthesis pathway.
